# LUKB: preparing local UK Biobank data for analysis

**DOI:** 10.1093/bioadv/vbae176

**Published:** 2024-11-09

**Authors:** Xiangnan Li, Yaqi Huang, Shuming Wang, Meng Hao, Yi Li, Hui Zhang, Zixin Hu

**Affiliations:** Shanghai Pudong Hospital, Fudan University, Shanghai, 201399, China; State Key Laboratory of Molecular Engineering of Polymers, Department of Macromolecular Science, Fudan University, Shanghai, 200438, China; Human Phenome Institute, Fudan University, Shanghai, 201203, China; Human Phenome Institute, Fudan University, Shanghai, 201203, China; Human Phenome Institute, Fudan University, Shanghai, 201203, China; Human Phenome Institute, Fudan University, Shanghai, 201203, China; Human Phenome Institute, Fudan University, Shanghai, 201203, China; Zhangjiang Fudan International Innovation Centre, Fudan University, Shanghai, 201210, China; Shanghai Pudong Hospital, Fudan University, Shanghai, 201399, China; Artificial Intelligence Innovation and Incubation Institute, Fudan University, Shanghai, 201210, China; Shanghai Academy of Artificial Intelligence for Science, Shanghai, 200240, China

## Abstract

**Motivation:**

The UK Biobank data holds immense potential for human health research. However, the complex data preparation and interpretation processes often act as barriers for researchers, diverting them from their core research questions.

**Results:**

We developed LUKB, an R Shiny-based web tool that simplifies UK Biobank data preparation by automating these preprocessing tasks. LUKB reduces preprocessing time and integrates functions for initial data exploration, allowing researchers to dedicate more time to their scientific endeavors. Detailed deployment and usage can be found in the Supplementary Data.

**Availability and implementation:**

LUKB is freely available at https://github.com/HaiGenBuShang/LUKB.

## 1 Introduction

UK Biobank is a treasure trove of medical data, encompassing genetics, proteomics, imaging, and medical records for over 500 000 participants (Sudlow *et al.* 2015, Miller *et al.* 2016, Bycroft *et al.* 2018, Gareth *et al.* 2024). This wealth of information has facilitated countless medical breakthroughs (Alayo *et al.* 2023, Dhindsa *et al.* 2023, Tian *et al.* 2023, Wang *et al.* 2024) and continues to fuel new discoveries (Supplementary Fig. S1). As research delves deeper into human health, utilizing UK Biobank data becomes increasingly crucial.

**Figure 1.**
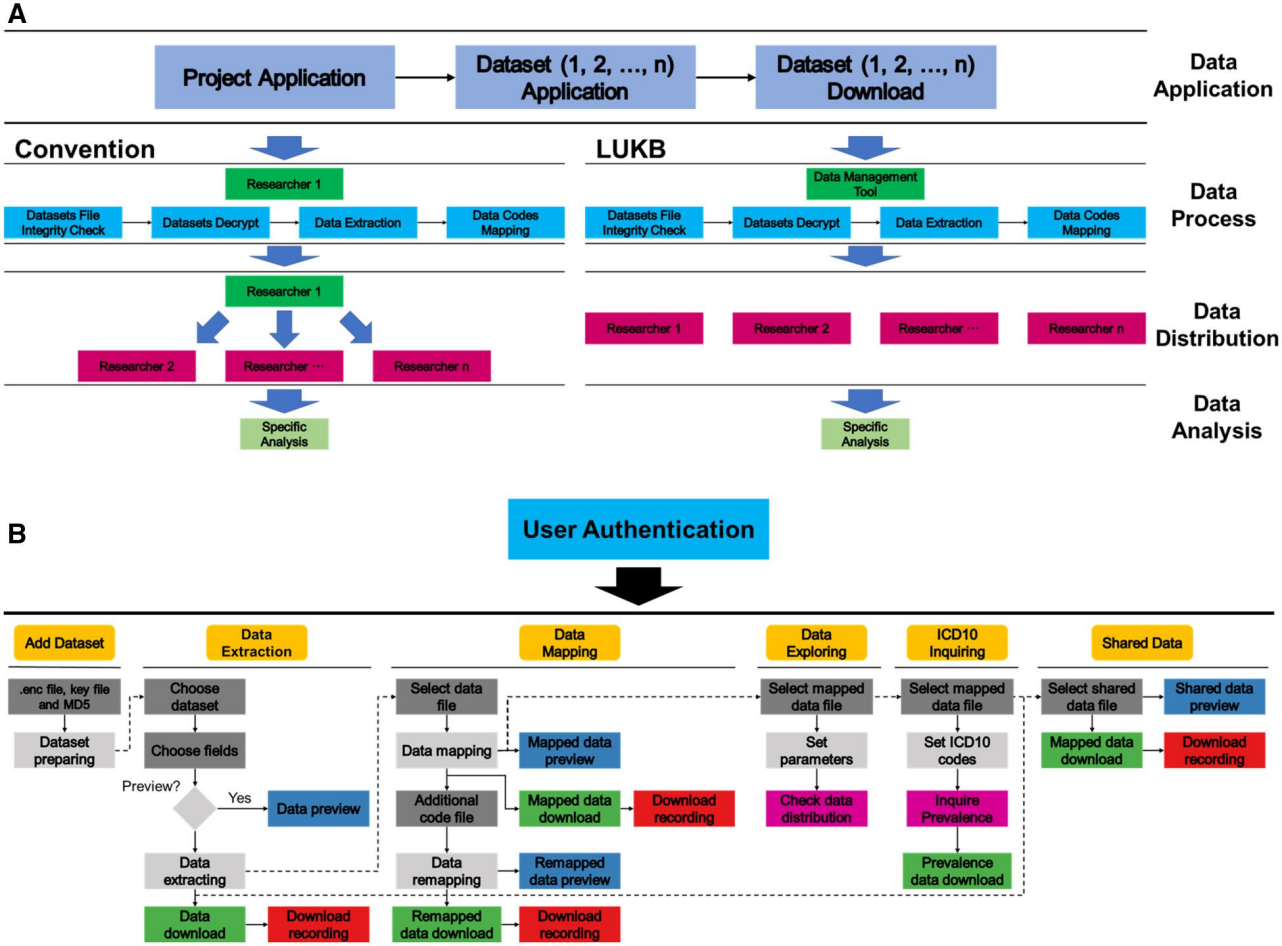
LUKB facilitates UK Biobank data preparation. (A) Steps for performing UK Biobank data analysis. The bottom-left side shows the conventional steps, while the bottom-right side illustrates the steps using LUKB. (B) LUKB data processing components and design.

However, navigating the vast data within UK Biobank can be challenging, posing obstacles to research speed and efficiency. Processing downloaded encrypted UK Biobank data often involves complex steps, potentially requiring multiple data applications and reprocessing if the data do not meet expectations (Fig. 1A). Although there are tools (Hanscombe *et al.* 2019, Pividori and Im 2019, Schneider-Luftman and Crum 2019, Kiral *et al.* 2020) available for dealing with UK Biobank data, they often skip these complicated steps. Moreover, data previews are unavailable before data processing, which might lead to frustration when the downloaded data proves unsuitable. While the UK Biobank Research Analysis Platform (RAP) allows data previewing, preparing and downloading the final data incurs costs, potentially restricting research scope. Despite the gradual transition to RAP, preprocessing steps remain inevitable for previously downloaded data.

In this article, we present LUKB, a freely deployable R Shiny-based web tool designed to simplify the preparation of UK Biobank data. LUKB incorporates utilities from the ukbtools package to facilitate initial data exploration, thereby maximizing the potential of UK Biobank data. Our locally deployed solution integrates data access, previewing, exploring, sharing, and ICD-10 inquiries within research groups. By providing instant data previews and streamlining the preparation process, LUKB minimizes unnecessary application steps and reduces the time and costs associated with UK Biobank data processing. This user-friendly tool empowers both experienced and novice researchers to unlock the full potential of UK Biobank data and accelerates the pace to life-changing discoveries.

## 2 Methods

### 2.1 Tool design

LUKB is designed to simplify access to UK Biobank data through seven key components (Fig. 1B), each addressing a specific stage of data preparation. ‘User Authentication’ safeguards data by filtering access, upholding ethical research practices. ‘Add Dataset’ effortlessly integrates downloaded data, streamlining workflows. ‘Data Extraction’ allows researchers to focus on their research by pinpointing specific fields of interest. ‘Data Mapping’ transforms raw data into their real-world meanings by deciphering cryptic codes and providing descriptive titles. ‘Data Exploring’ enables researchers to generate summary figures of the distribution of a specific field for a subset of individuals relative to a reference set from the mapped data. ‘ICD10 Inquiring’ provides the prevalence for different diseases and returns the prevalence of stratified populations by levels of a reference variable in the UK Biobank cohort based on given ICD-10 codes. Finally, ‘Shared Data’ fosters collaboration by securely storing files for exchange between colleagues, accelerating scientific breakthroughs.

#### 2.1.1 User authentication

LUKB prioritizes responsible data management by ensuring that only authorized researchers can access UK Biobank data. A text file securely stores usernames and encrypted passwords using the scrypt algorithm, allowing data holders to easily manage access. This secure setup safeguards sensitive information while remaining user-friendly for adding or removing researchers.

#### 2.1.2 Add dataset

This component handles encrypted UK Biobank data (.enc files) with ease, seamlessly decrypting them using corresponding key files (.key files) and verifying their integrity through MD5 string checks. Once decrypted, the data are passed to the ‘Data Extraction’ component. Simultaneously, a comprehensive dictionary is generated, containing field descriptions, item counts, and data-coding indices to guide researchers through the dataset’s structure.

#### 2.1.3 Data extraction

After data decryption, researchers can preview and extract specific data fields of interest. LUKB enables users to preview the first 100 records or extract the data directly. To optimize performance and prevent tool unresponsiveness, the data extraction tasks are submitted to the operating system, circumventing R’s single-thread limitation. Once the extraction task completion signal is detected, the extracted data are passed to the ‘Data Mapping’ component for download. To further protect data, download recording is triggered for all download actions, aiding in the identification of any abnormal downloads.

#### 2.1.4 Data mapping

The extracted data from some fields is less readable for humans as it consists solely of codes and numbers. To enhance readability, it is essential to transform these codes into their real-world meanings. UK Biobank provides multiple code mapping files to assist in this process. This component leverages the most comprehensive code mapping file to perform the transformation, providing instant previews of the mapped data. However, in cases where certain fields remain unmapped, researchers could upload additional coding files for those specific fields, ensuring a comprehensive mapping of all available data.

#### 2.1.5 Data exploring

Researchers can explore the data distribution as a preliminary stage of analysis. This component extends the summarization methods from ukbtools to cover all tabular data in the UK Biobank. By selecting an variable of interest and another variable to stratify the individuals into a subset and a reference set, researchers can examine the distribution across the subsets and reference sets (Supplementary Fig. S7).

#### 2.1.6 ICD-10 inquiring

ICD-10 inquiring allows researchers to investigate the prevalence of diseases in the UK Biobank cohort by providing the relevant ICD-10 codes. Disease cases are counted, and the prevalence can be stratified by a reference variable, enabling comparisons across different populations, a method adopted from ukbtools (Supplementary Fig. S8).

#### 2.1.7 Shared data

The Shared Data component enables researchers to share data files securely within a research group. Each shared file requires a brief comment, enabling clarity for collaborators when accessing those files.

### 2.2 Large dataset handling

UK Biobank datasets can be extremely large, with the size of decrypted data increasing by up to 5 times compared to the original encrypted data file. Loading an entire decrypted dataset into main memory can be inefficient and resource-intensive. To address this, LUKB does not process the entire decrypted dataset directly but enables users to select definite fields for extraction and analyzing. This approach optimizes performance and allows for efficient downstream analysis with a manageable dataset.

## 3 Results and discussion

LUKB is designed primarily to simplify the data preparation steps of UK Biobank spreadsheet data and serves as a data exploring tool for data distribution and ICD-10 prevalence. Compared to UK Biobank RAP, LUKB requires no additional costs, is easier to use, and saves computational resources when compared to ukbtools.

### 3.1 LUKB simplifies the preparation of analysis-ready data from local UK Biobank data

Traditionally, accessing and preparing UK Biobank data for analysis involves a complex sequence of applications, downloads, and manual processing. LUKB significantly streamlines this process, transforming local UK Biobank data into analysis-ready data with just a few actions (Fig. 1A). Previously, researchers had to download data to local computers after a successful data application, then navigate multiple steps like data integrity checks, decryption, specific data extraction, and code mapping. Sharing these data often required physical devices like hard drives or UKB flash drives. This process, repeated for each new data application, was both tedious and time-consuming. LUKB eliminates these complexities, allowing researchers to bypass the time-consuming steps and focus on discoveries.

### 3.2 Case study: preparing data for disease trajectory analysis

**Figure 2.**
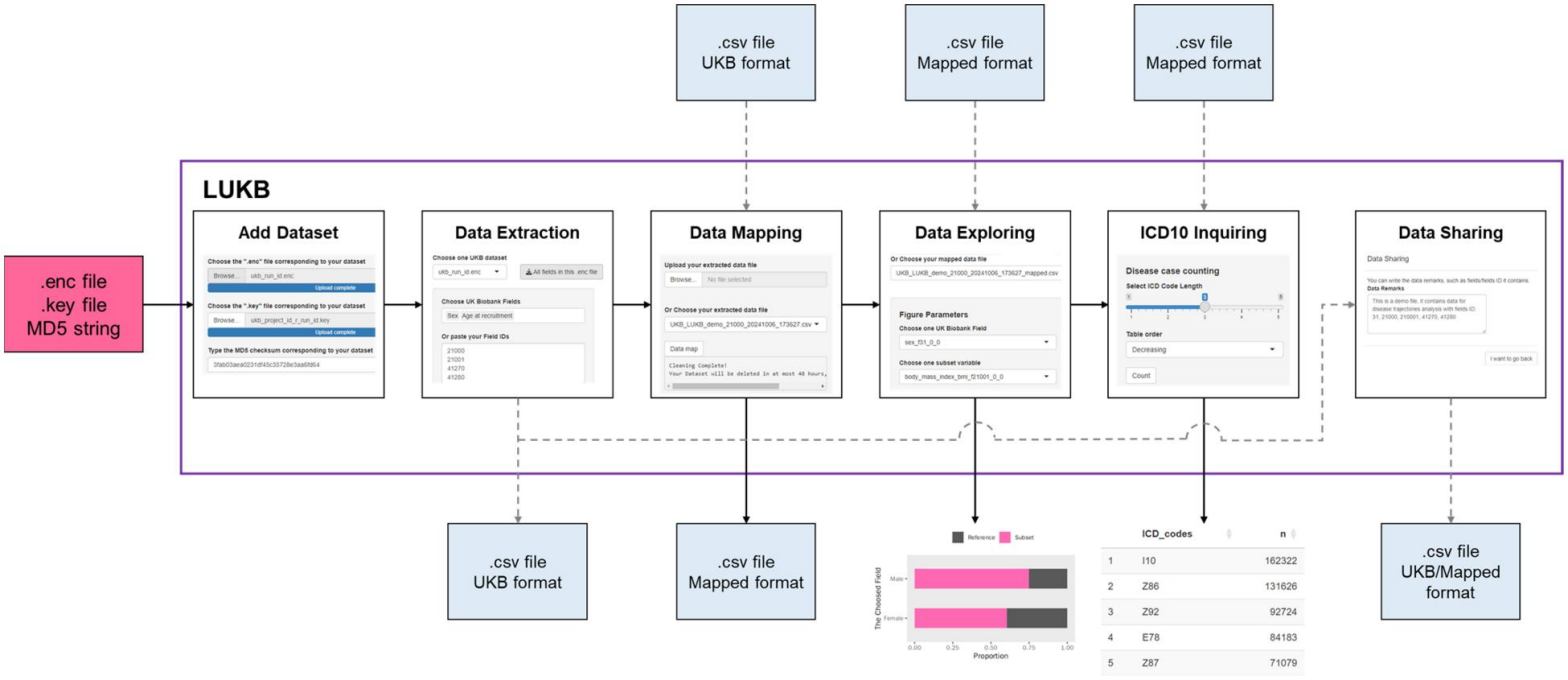
LUKB data processing workflow. LUKB requires UK Biobank encrypted data file (.enc), decrypted key (.key), and MD5 string as inputs. After importing the data, researchers can extract specific data by selecting field names and providing field IDs. The data can then be mapped to facilitate interpretation. LUKB supports exploring data distribution and ICD-10 prevalence. All extracted and mapped data files can be shared through LUKB. For Data mapping, Data exploring, and ICD10 inquiring components, uploading data files with UK Biobank header format or the mapped header format generated by LUKB is supported. Black solid arrows indicate the processes performed in the case study, while grey dash arrows indicate the processes supported by LUKB.

LUKB streamlines the process of preparing local UK Biobank data and requires three key inputs for data preparation: the downloaded UK Biobank encrypted data file (.enc), its corresponding decryption key (.key) and the MD5 string for the encrypted file. This case study demonstrates how LUKB handles data preparation for disease trajectory analysis, which involves information about sex, age, ethnics, body mass index (BMI), ICD-10 diagnoses, and corresponding diagnosing time (Field ID: 31, 21022, 21000, 21001, 41270, and 41280) of each participant. The main steps are outlined below (Fig. 2), with additional details available in the Supplementary Data.

#### 3.2.1 Import UK Biobank data into LUKB

To begin the data preparation process, users upload the downloaded encrypted UK Biobank data file (.enc), the corresponding key file (.key), and the MD5 string to LUKB after logging in in the “Add Dataset” tab. Once the upload is complete, the data preparation task can be initiated by clicking “Start preparing your data,” with task status tracked via the log file.

#### 3.2.2 Extract raw spreadsheet data

After the data are successfully imported, researchers can extract specific fields for further analysis. In this case, we selected and provided fields for sex, age, BMI, ICD-10 diagnosis, and diagnosis dates. After previewing the data to confirm correctness, the extraction process was started by clicking the “Extract data!”. The extraction task status is updated every 5 s, and once completed, the extracted data are available for download or further processing via the “Download” button.

#### 3.2.3 Map data for disease trajectory analysis

The extracted UK Biobank often contains coded files that are not immediately interpretable. The “Data Mapping” component decodes these fields, converting them into meaningful real-world labels. After selecting the extracted data file to map, the mapping process is initiated by clicking “data map” button. The mapped file can be previewed in the right panel. After mapping, the data for disease trajectory were ready.

#### 3.2.4 Explore data characteristics

Before proceeding with the analysis, researchers can explore key characteristics of the data to ensure data integrity and avoid potential biases. For example, using LUKB, researchers can examine BMI distributions across different demographic groups, such as comparing BMI ≥25 between males and females. This step ensures that the data are well-prepared for unbiased downstream analysis.

#### 3.2.5 Inquire prevalence of ICD-10 diseases

In addition to data extraction and mapping, LUKB supports users in preparing datasets by providing summary statistics related to ICD-10 disease prevalence. Researchers can use this feature to quickly summarize the number of cases for specific diseases within the UK Biobank cohort by ICD-10 codes, making it easier to determine which data subsets should be further analyzed. For example, hypertension (I10) and lipoprotein metabolism disorders (E78) were identified as highly prevalent in the dataset. These summaries help researchers efficiently organize their data before conducting in-depth analyses using external tools.

### 3.3 Comparison to UK Biobank RAP and ukbtools

While LUKB, UK Biobank RAP, and ukbtools can serve as tools for UK Biobank data preparation and analysis, they are different in their primary purpose, features, and accessibility (Table 1 and Supplementary Table S1).

**Table 1.** Key feature differences among LUKB, UK Biobank RAP, and ukbtools.

Feature	LUKB	UK Biobank RAP	ukbtools
Type	Web tool	Web tool	R package
Purpose	Comprehensive data preparation	Commercial data analysis platform	Data preparation for downstream steps
Data import	Automatic decryption (.enc, .key, MD5)	Automatically assigned on platform	Manual decryption and conversion
Data extraction	Batch field selection with preview	Manual field selection, separate preview	Not available
Data Mapping	Automatic with custom mapping support	Only mapped or unmapped data can be access, no partial mapping	Only header mapping
Cost	Free	Costs for data storage, extraction, and download	Free

LUKB is designed to simplify the entire data preparation process from start to finish, offering an intuitive web-based interface that automates key steps such as data import, extraction, and mapping. It particularly facilitates data communication within researcher groups. In contrast, UK Biobank RAP is a commercial platform that focuses on data analysis but incurs costs for data storage, extraction, and downloads. ukbtools is an R package that primarily supports downstream data analysis but requires manual decryption and data conversion before use.

LUKB stands out for its ability to handle batch field selection with real-time data preview, seamless collaboration within research groups, and free access and local deployment, which avoids the additional costs associated with UK Biobank RAP.

### 3.4 Benchmark

The performance of LUKB for data preparation varies depending on the complexity of the dataset being prepared. For example, importing a dataset with 671 fields takes 37 s, while a dataset with 2354 fields, the 14 core categories, takes 96 min. For data extraction, LUKB can extract all data from a 671-field dataset in 58 s, while extracting 6 fields for disease trajectory analysis from the 2354-field dataset takes 71 min.

In comparison, UK Biobank RAP extracts the same data totally in 18 min at a cost of £0.0201. However, RAP requires users to manually add each field individually, resulting in at least 269 “select and click” actions, which requires additional researcher labor time, whereas LUKB allows users to provide all required fields in a single action.

Benchmark tests for LUKB were conducted on an Intel(R) Core(TM) i9-9900K CPU @ 3.60 GHz, 32G RAM server and for RAP on an instance mem1_ssd1_v2_x4.

### 3.5 Limitations and recommendations

LUKB has two main limitations: it cannot handle UK Biobank bulk/genomic data and its processing time increases when dealing with large datasets. While valuable, bulk/genomic data, such as images and raw sequencing data, require specialized tools for analysis due to their unstructured formats. Therefore, LUKB focuses on the majority of tabular, non-bulk/genomic, data, ensuring a familiar and accessible format for researchers. Additionally, for large datasets, LUKB’s single-threaded task processing can be time-consuming, which affects its efficiency.

To mitigate these limitations, we recommend using LUKB for non-bulk/genomic data. For very large datasets, researchers could break them down into small subsets to improve processing time. Deploying LUKB on a server with encrypted traffic within an intranet can further enhance data security and help avoid external attacks from the internet.

## 4 Conclusion

We have developed LUKB to facilitate researchers in making meaningful discoveries by simplifying complex data preprocessing and exploration steps into straightforward actions. LUKB also serves as an alternative to the UK Biobank RAP for preparing UK Biobank data, especially for budget-limited research groups. Although UK Biobank is gradually transitioning to the RAP for data preparation, LUKB remains compatible with the raw UK Biobank data prepared by RAP.

As an open-source tool, LUKB has the potential to integrate more personalized analysis methods, further accelerating and expanding the discoveries made using UK Biobank data, without incurring additional costs for researchers, something less feasible with RAP due to its commercial nature. Additionally, given the relatively small size of UK Biobank tabular data, even a modest workstation can handle the computational demands. Therefore, future enhancements could include building more advanced analysis functions within LUKB for local computer deployment, which could be particularly beneficial for researchers with limited coding experience.

In conclusion, our R Shiny-based LUKB empowers researchers with streamlined UK Biobank data preparation and initial exploration, enabling efficient analyses and potentially groundbreaking research advancements. By simplifying the data handling process, LUKB allows researchers to focus more on their scientific questions.

## Supplementary Material

vbae176_Supplementary_Data

## Data Availability

The data underlying this article are available in UK Biobank at https://www.ukbiobank.ac.uk/ and can be applied for through the UK Biobank Access Management System (AMS).
