# Supplementary material for: LUKB: preparing local UK Biobank data for analysis
Source: Bioinform Adv. 2024 Nov 9;4(1):vbae176. doi: 10.1093/bioadv/vbae176 (PMC11580680; doi:10.1093/bioadv/vbae176)
Supplement: vbae176_Supplementary_Data [file vbae176_supplementary_data.docx]

**Supplementary Data**





**Figure S1. Publications related to “UK Biobank” have rapidly increased in recent years.** Figure S1 shows the publications indexed by Web of Science under the topic of “UK Biobank”, reflecting the rapid growth in research involving this valuable resource.

**Table S1. Feature Differences among LUKB, UK Biobank RAP, and ukbtools**

| Feature | LUKB | UK Biobank RAP | ukbtools |
| --- | --- | --- | --- |
| Type | Web tool | Web tool | R package |
| Purpose | Comprehensive data preparation | Commercial data analysis platform | Data preparation for downstream steps |
| Data import | Automatic decryption (.enc, .key, MD5) | Automatically assigned on platform | Manual decryption and conversion |
| Data extraction | Batch field selection with preview | Manual field selection, separate preview | Not available |
| Data Mapping | Automatic with custom mapping support | Only mapped or unmapped data can be accessed, no partial mapping | Only header mapping |
| Data exploring | Data Distribution and detailed ICD-10 prevalence | Data Distribution and detailed ICD-10 prevalence | Data Distribution and detailed ICD-10 prevalence |
| Data Sharing | Through network traffic | Through network traffic with costs for data storage and download | Not available |
| Performance | Dependent on dataset size; slower for large datasets | Fast data extraction in “Dashboard” mode | Computing resource consumption when dealing with large datasets |
| Cost | Free | Costs for data storage, extraction, and download | Free |

**LUKB Deployment and Usage**

1. **Dependencies**

LUBK was developed using R version 4.2.3, so it is recommended to install R 4.2.3 or higher. Some required packages can be installed by executing the following command at the main directory after downloading the source code:

*Rscript required_packages.R*

To deploy LUKB on a server with encrypted network traffic, Nginx or other reverse proxies, Shiny Server, and a Secure Sockets Layer (SSL) certificate are required. To facilitate deployment of LUKB within Nginx, a Nginx configuration is provided.

1. **Add Users and Start LUKB**

LUKB will start automatically after user information is added. LUKB uses a text file to store user information. The passwords are encrypted using the scrypt algorithm. After downloading the source code, change to the main directory, and then execute:

*chmod +x add_users.sh && ./add_users.sh*

Then type the user name, password, and TRUE or FALSE to set data file download permission (Figure S2). LUKB will start in a short time.

If using Nginx and Shiny Server, ensure that the port used by Shiny Sever is allowed through the firewall. To allow access to the default Shiny Server port (3838) on Ubuntu, execute the following:

*sudo iptables -I INPUT -p tcp --dport 3838 -j ACCEPT* (Ubuntu)

This step is not needed for CentOS. Then researchers can access LUKB via https://your_server_ip/LUKB/ if Nginx and Shiny Server are configured correctly.

If Nginx and Shiny Server are not being used, the default port used by LUKB (1111) should be added to the firewall rules. To allow access to port 1111, execute:

*sudo iptables -I INPUT -p tcp --dport 1111 -j ACCEPT* (Ubuntu)

or

*sudo firewall-cmd --add-port=1111/tcp* (CentOS)

The port 1111 will be opened temporarily. Then researchers can access LUKB via http://your_server_ip:1111/. To change the default port, change “port = 1111” to “port = the_port_you_want” on line 10 of launch_app.sh file.


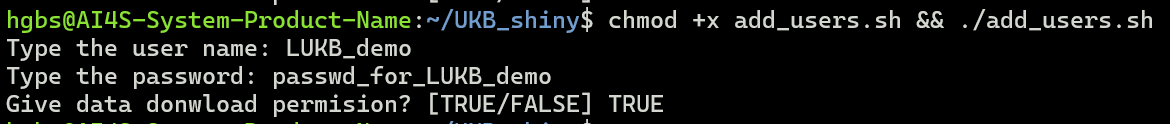


**Figure S2. Add user information.**

1. **Access to LUKB**

As a web-based tool, LUKB is easily accessible. To access LUKB, open the link https://your_server_ip/LUKB/ or http://your_server_ip:1111/ in the web browser (**Figure S3**). Once authenticated, researchers can access data through the user interface.

**
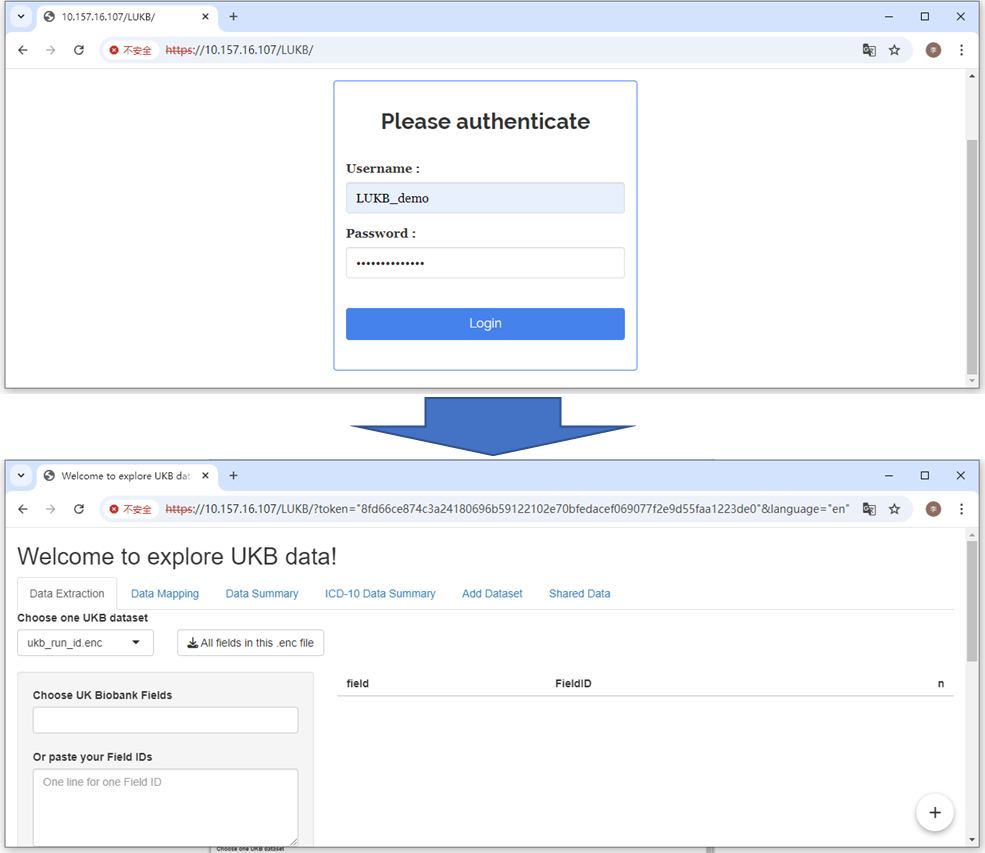
**

**Figure S3. Access to LUKB.** By opening the link (*https://your_server_ip/LUKB/ or http://your_server_ip:1111/*) in the web browser, researchers can access to LUKB.

1. **Add Dataset**

For initial use, researchers must import UK Biobank data into LUKB. Typically, UK Biobank data is downloaded in a file named *ukbrun_id.enc*, where *run_id* corresponds to your specific data basket. This file is accompanied by a key file and an MD5 checksum. To add data to LUKB, researchers should upload the downloaded *.enc* file, the corresponding key file, and provide the MD5 string. Once these are entered, the data will be imported into LUKB (**Figure S4**).


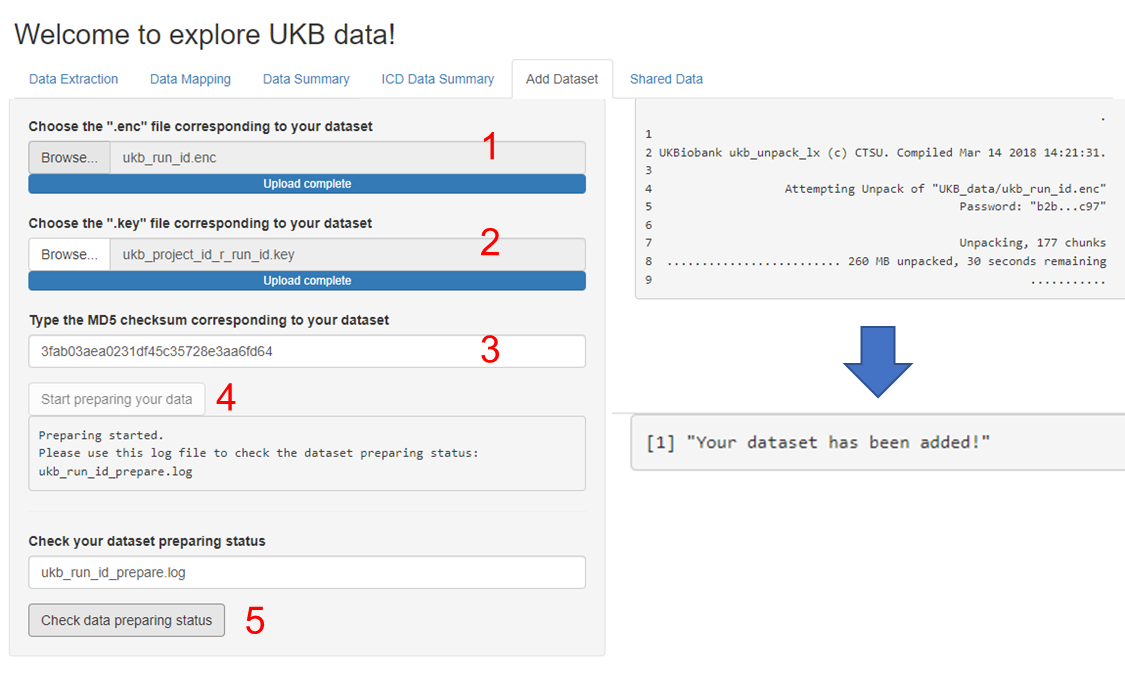


**Figure S4. Add dataset to LUKB.** Researchers can add UK Biobank datasets to LUKB by providing the .enc file, key file, and MD5. The right panel displays the data preparation status upon clicking the “Check data preparation status” button. The message “Your dataset has been added!” indicates successful dataset addition to LUKB. 1) upload *.enc* file. 2) upload key file. 3) provide MD5 string. 4) submit dataset adding task to operating system. 5) check dataset adding task status.

1. **Data Extraction**

Once data importation is complete, researchers can extract data of interest by selecting specific fields or providing field IDs. LUKB also allows researchers to preview the data of these selected fields before extraction. To reduce the data extraction burden, each user can submit only one data extraction task one time. The data extraction status will be monitored in the “Status” section. After data extraction is complete, researchers with data download permission can download the extracted data (**Figure S5**).


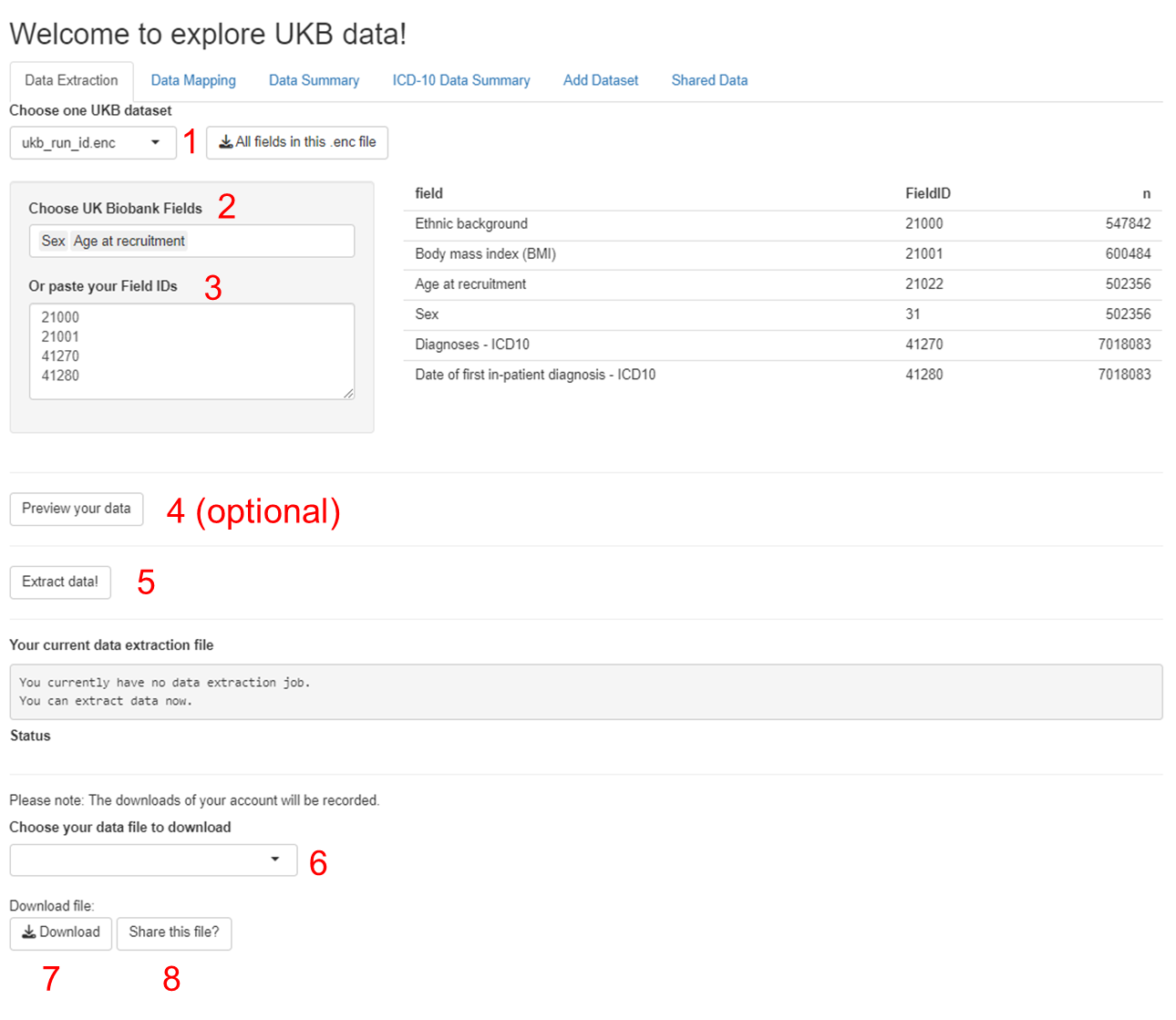


**Figure S5. Extracting Data from an Imported Dataset.** After selecting a dataset, researchers can either choose specific fields or provide field IDs to extract the data of interest. 1) choose the dataset for extraction. 2, 3) provide fields or field IDs for data extraction. 4) preview the data of provided fields (optional). 5) submit data extraction task to operating system. 6) choose a data file to be downloaded. 7) download the extracted data. 8) choose to share the extracted data file.

1. **Data Mapping**

In some cases, extracted data may not be directly readable due to the use of codes, such as in the field “sex” (Field ID: 31). For example, “Male” might be represented as “1” in this field. To enhance readability, LUKB allows researchers to map these codes to their real meanings. To perform code mapping, click the “Data Mapping” button after uploading or choosing a data file. After mapping, researchers can preview the remapped data. However, some fields might still be unmappable. This typically occurs when the mapping information is stored in separate coding files. For instance, the coding information for “Non-cancer illness code, self-reported” (Field ID: 20002) is stored in the Data-Coding 6 file. To map these fields, researchers need to upload the relevant coding files and specify the field to be mapped (**Figure S6**).


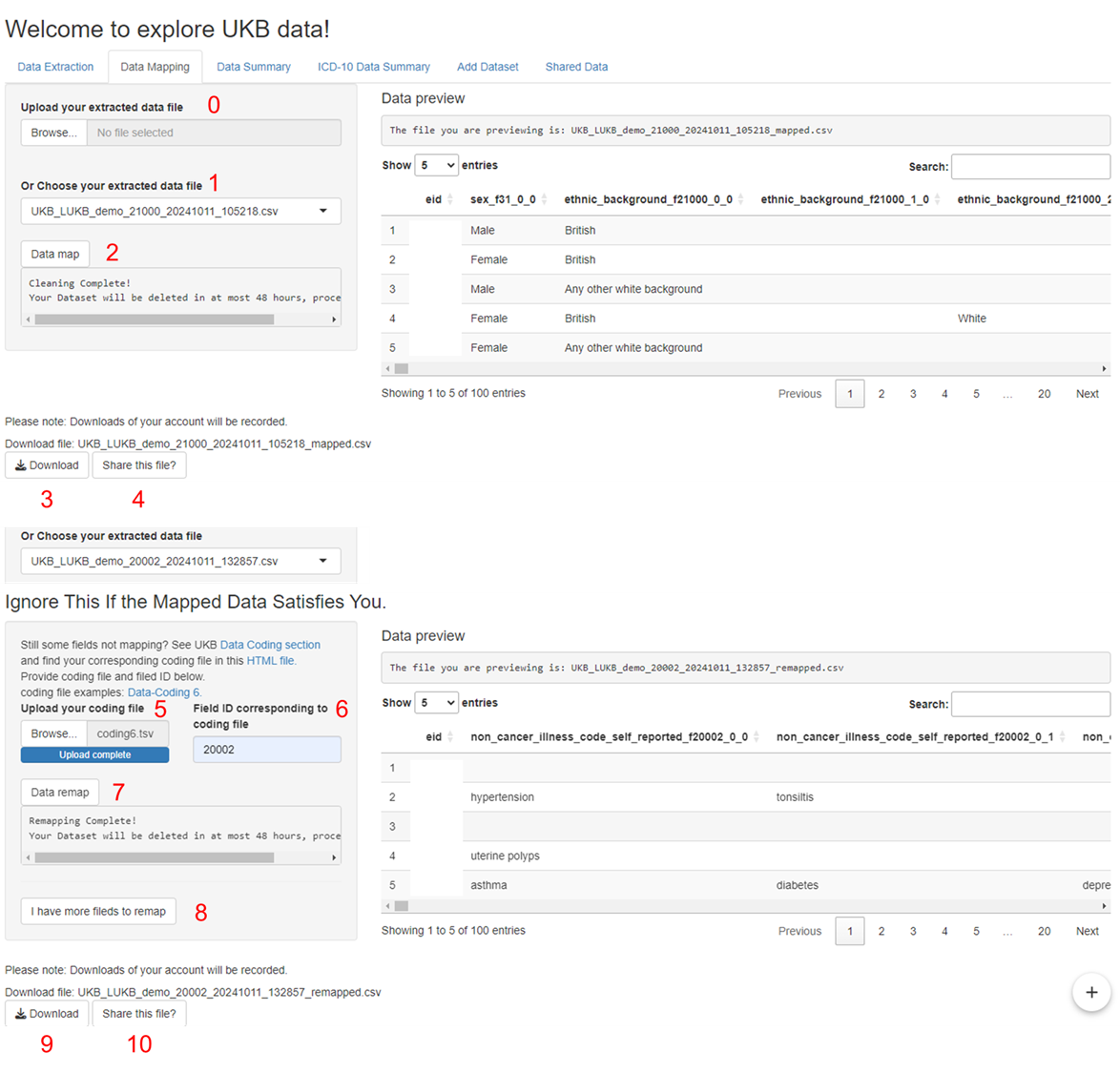


**Figure S6. Mapping codes to their real meanings.** 0) upload data file produced in Data Extraction component. 1) choose the file for code mapping. 2) submit code mapping task. 3) download the mapped data file. 4) choose to share the mapped data file. 5) upload relevant data coding file. 6) specify the field ID corresponding to the coding file. 7) submit code remapping task. 8) optionally, add more coding file and fields to map, this step can be repeated multiple times. 9) download the remapped data file. 10) choose to share the remapped data file.

1. **Data Exploring**

For the mapped file, researchers can explore the data distribution by selecting a variable of interest and a stratifying variable to stratify the individuals. For numeric stratifying variables, the subset is defined as individuals with values larger or equal to the threshold of the stratifying variable, while the reference set comprises individuals with values below the threshold. For categorical stratifying variables, the subset includes individuals with values matching the threshold of the stratifying variable. Other parameters can be adjusted to refine the output figure (**Figure S7**).


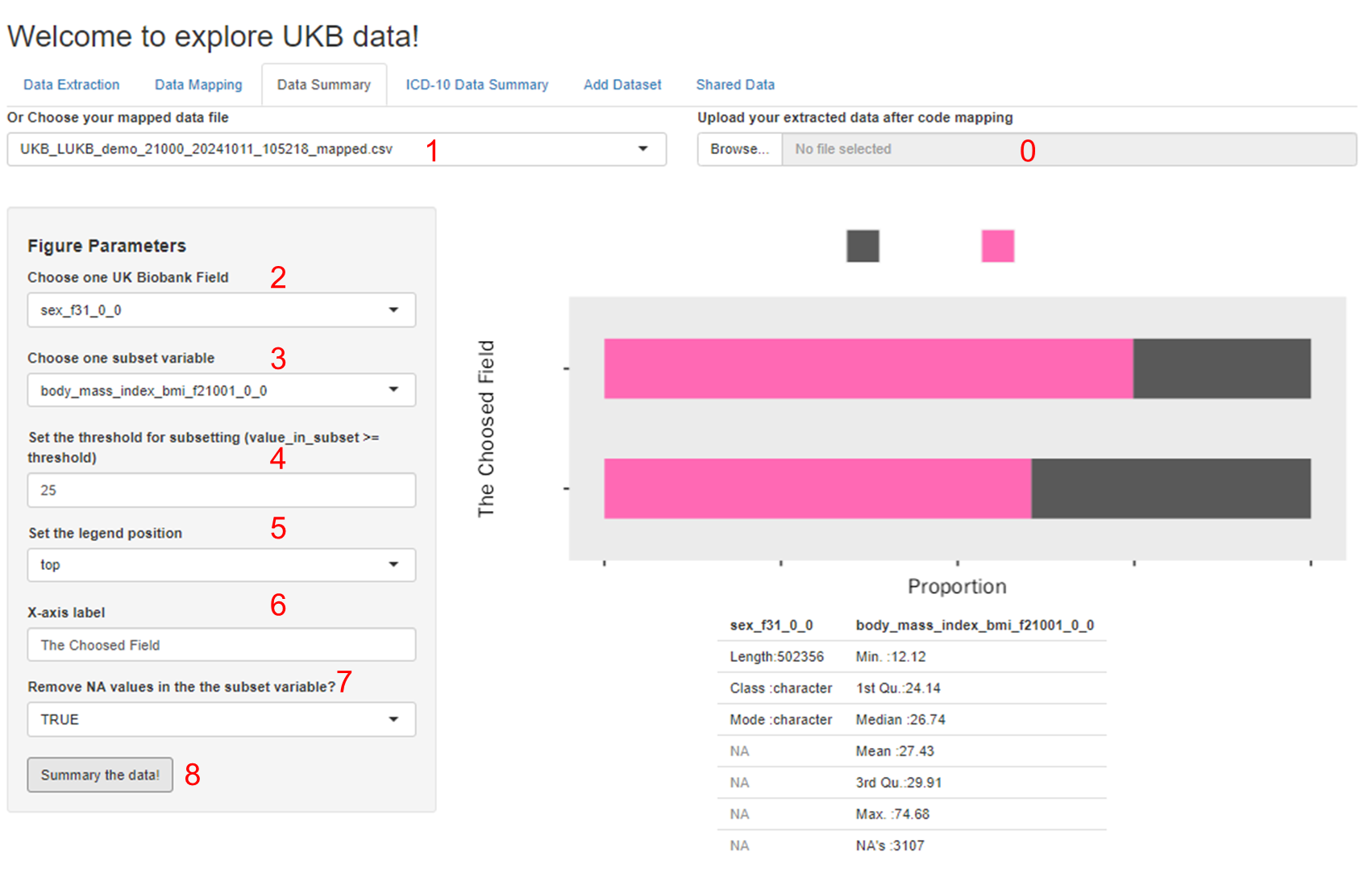


**Figure S7. Exploring data distribution.** 0) upload data file produced in Data Mapping component. 1) choose the file to explore the data distribution. 2) select the variable of interest. 3) select a stratifying variable. 4) set the threshold to stratify the individuals into a subset and a reference set. 5) choose the position of the legend. 6) specify the label for x-axis. 7) indicate whether to remove the individuals with NA values in the stratifying variable. 8) generate the figure and summarize the two variables.

1. **ICD10 Inquiring**

For the mapped data files containing ICD-10 diagnosis data, the prevalence of diseases can be summarized by providing the corresponding ICD-10 codes. This includes the count of cases for each disease. By specifying the ICD-10 prefix length, cases corresponding to each ICD-10 code can be counted. By entering specific ICD-10 codes or their prefixes, the prevalence of related diseases in the UK Biobank cohort can be summarized. Additionally, by providing a reference variable, the prevalence of different diseases can be compared according to the levels of the reference variable (**Figure S8**).


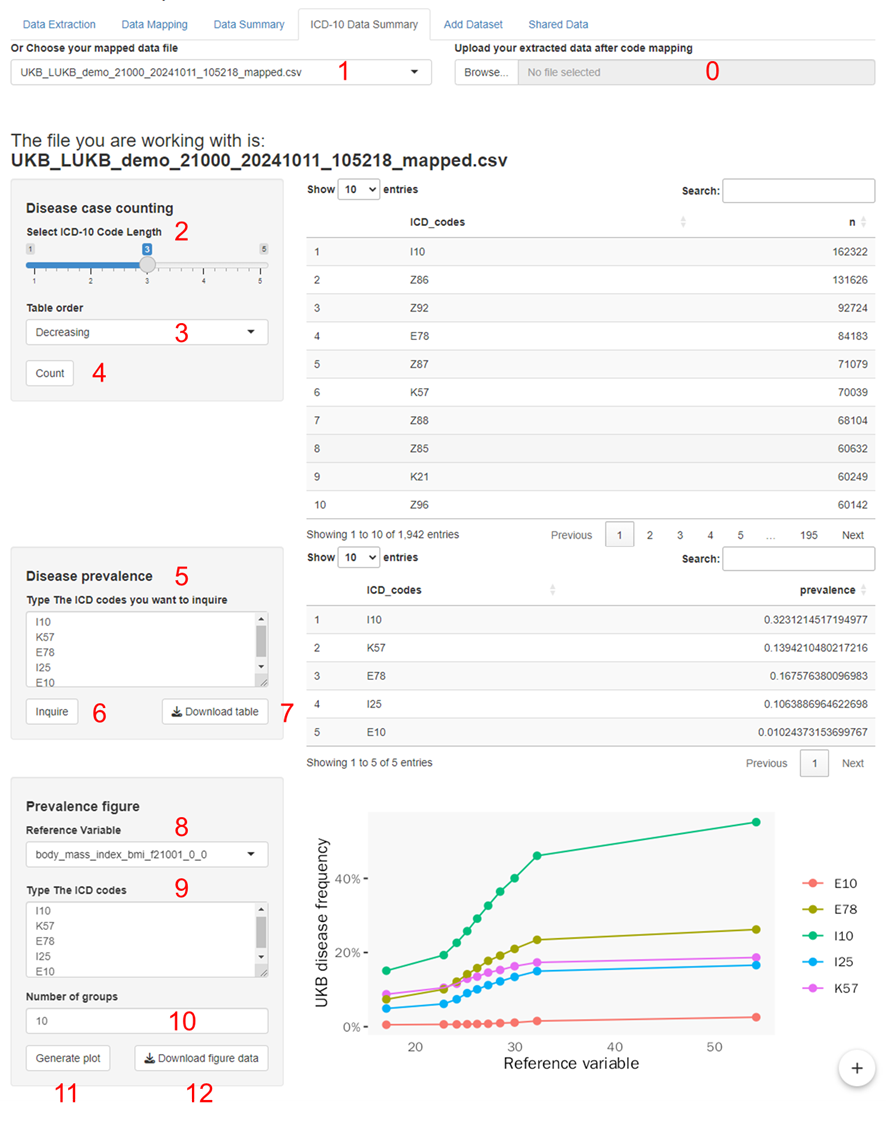


**Figure S8. Summarizing the cases and prevalence of different diseases for the UK Biobank cohort.** 0) upload data file produced in Data Mapping component. 1) choose the file to summarize the prevalence. 2) select the length of ICD-10 codes to count the cases of different diseases (e.g., length 1 for “I”, length 2 for “I10”). 3) set the display order of the disease case count table. 4) click to count disease cases. 5) enter the ICD-10 codes or prefixes to summarize the prevalence of corresponding diseases. 6) click to summarize the prevalence. 7) download the summarized prevalence table. 8) select a reference variable to compare the prevalence of different diseases. 9) enter the ICD-10 codes or prefixes of disease to be compared. 10) set the number of groups stratified by the values of the reference variable. 11) click to produce the figure. 12) download the data that generates the figure.

1. **Data Sharing**

LUKB encourages researchers to share extracted or mapped data with others, as unshared files are automatically removed after a maximum of 48 hours to conserve system storage. Upon completing data extraction or mapping, researchers can share a file by clicking the “Share this file?” button. Adding optional remarks about the file’s contents can help other researchers quickly familiarize themselves with the shared data. All shared files can be viewed and downloaded within the “Shared Data” component (**Figure S9**).


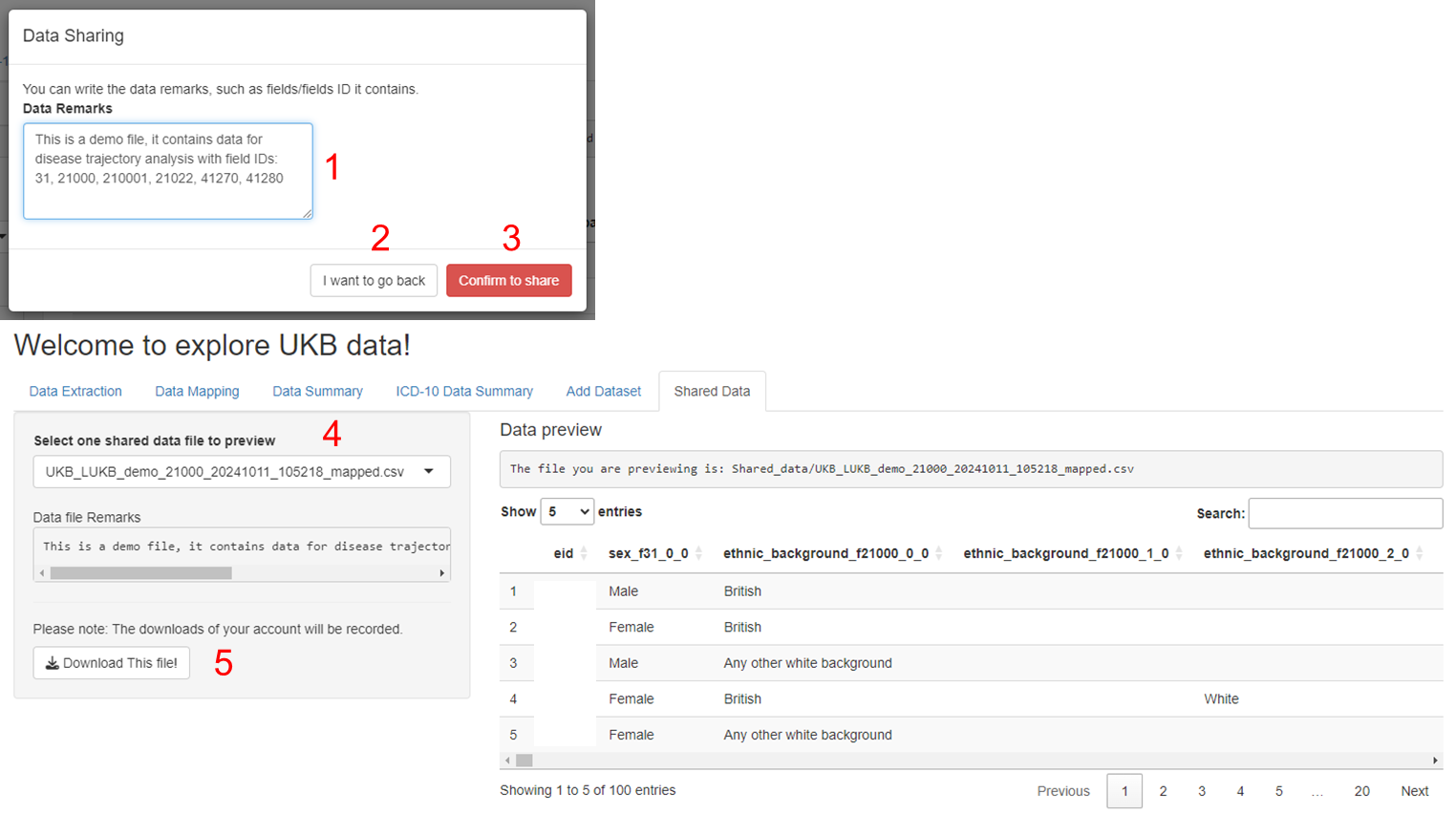


**Figure S9. Shared data file remarks and download.** Each shared data file is accompanied by a descriptive file providing context and insights. When choosing to share a file, researchers should provide a descriptive overview. 1) description information. 2) cancel data file sharing. 3) confirm data file sharing. 4) choose one shared data file. 5) download the shared data file.

1. **Downloading Monitor**

LUKB maintains comprehensive records of user downloading activities for tracking and auditing purposes. These records, including downloaded files, specific fields extracted, and corresponding user information, can be found within the "Logs/" directory.
